# Supplementary material for: Environmental drivers of stream metabolism in a middle TN headwater stream
Source: PLoS One. 2024 Dec 31;19(12):e0315978. doi: 10.1371/journal.pone.0315978 (PMC11687656; doi:10.1371/journal.pone.0315978)
Supplement: S1 File — S1.1 MiniDOT vs EXO2. S1.2 HOBO Light Logger vs LI-1500 Light Sensor Logger. S1.3 HOBO Light Logger Orientation. S1.4 Consistency of MiniDOT Measurements. (DOCX) [file pone.0315978.s001.docx]

## **S1 Sensor Precision Tests**

A goal of this study was to test the precision and reliability of new light and dissolved oxygen sensors and see if they can make continuous stream metabolism measurements at a lower cost. We designed a few experiments to compare the precision of more affordable sensors such as MiniDOTs and HOBO Pendant MX Temperature/Light Data Loggers with DO measurements made using a YSI EXO2 and PAR measurements made using a LI-COR LI-192 underwater quantum sensor with a LI-1500 Light Sensor Logger. These tests showed that the MiniDOTs and HOBO Pendant MX Temperature/Light Data Loggers serve well for the purpose of this study.

### S1.1 MiniDOT vs EXO2

In July 2022, we deployed one MiniDOT and one EXO_2_ together at Site 1 to make continuous DO measurements every 5 minutes over four days. The measurements from the two sensors have a linear relationship with R^2^ value of 0.995 and a y-intercept at 0 with a slope of 0.9087. This relationship shows that the two pieces of equipment have consistent measurements but EXO_2_ will have slightly higher DO measurements. It might be caused by the differences in the equipment’s physical design. For EXO_2_, all the sensors are put inside a half-open shield to protect them from potential blockages such as leaves and mud. The sensor for MiniDOT is directly exposed to the environment and can be blocked sometimes. In our field measurements, we set the MiniDOT pointing downstream so there is little chance for the sensor to be blocked. Starting in October 2022, we deployed Miniwiper together with MiniDOT so that the sensor is constantly cleaned in the field, further reducing the chance of potential blockage. Despite the differences in DO measurements, the BASE model outputs calculated using these two DO measurement sets also showed good agreement and NEP is always negative. The differences between GPP values are less than 10%, between ER values are less than 30%, and between K_O2_ values are less than 10%, which are acceptable for our purpose of identifying the seasonal and diurnal variations.

### S1.2 HOBO Light Logger vs LI-1500 Light Sensor Logger

In October 2022, we tested the precision of the HOBO Light Logger by comparing its measurement with the LI-COR LI-192 Light Sensor’s measurement. We set both loggers outside on Vanderbilt University campus for 36 hours. After converting HOBO raw data to PAR by multiplying the converting factor 0.0185, the result shows good agreement with the data measured by LI-192 at log scale, but in linear scale the relationship becomes nonlinear when lux readings were above 4324 lux. In our measurements, there was a maximum value of around 40000 lux, so using the HOBO Light Logger may lead to inaccuracy in the result. However, in EFC, when the sensor is not directly exposed to sunlight, in a cloudy day or in the shade, the light logger readings are in the range where HOBO Light Logger and LI-192 shows good agreement.

In June 2023, we did another test to see if underwater measurements from HOBO Light Logger and LI-192 would still show good agreement. In the field experiment, we placed the two loggers underwater and made simultaneous measurements. The results were similar with the October 2022 experiment. In total, we made 22 measurements, 2 from EFC site 1, 1 from EFC site 2, and 19 from Stephens Lake. After converting HOBO data to PAR values by multiplying the same converting factor, LI-192/HOBO averaged over all measurements is 1.2, but the standard deviation is 1.3 which is high. The data distribution is consistent with our previous measurement on Vanderbilt University campus. In this test, LI-192/HOBO has a stable value around 0.67 when HOBO measurement is below 7000 lux, but the ratio starts to show large variations with the range from 0.3 to 1.7 when the light intensity is above 7000 lux.

### S1.3 HOBO Light Logger Orientation

In April 2023, we tested how the orientations of the HOBO Light Logger impact its light intensity measurement under the water and how this difference influences the metabolism calculation. In this experiment, we deployed 4 HOBO Light Loggers at Stephens Lake together, three under the water and one on the deck. Although the measurements were made at the lake, the results have implications for our stream measurements made using the same sensors. The three loggers under the water were facing up, sideways, and down to measure the light from different directions and to simulate three ways to deploy the HOBO Light Logger in the field. The underwater sensors were 10 inches below the water surface. As expected, the logger received much less light when pointed down because the body of the logger blocks overhead light from the sensor. The logger pointing sideway had the measurement closer to the logger facing up but still lower. Consequently, we had lower GPP estimations when used the measurement from the logger facing sideway. Hence, in future deployments, it is important to keep the HOBO Light Logger in the “up” position, which we attempted to do in all our field deployments.

S1.1 Fig. Measured light intensity for four HOBO light loggers. One was facing up in the air, and three were deployed 10 inches below the water surface, one up, one down, and one sideways.

### S1.4 Consistency of MiniDOT Measurements

In September 2023, we deployed two MiniDOTs, #786242 and # 792188 at Site 2 to test if they would make consistent measurements since we had been alternating them during our field campaign. Their measurements showed a good linear agreement with R^2^ value of 0.9984, and the BASE model outputs are also similar (Fig S1.1).


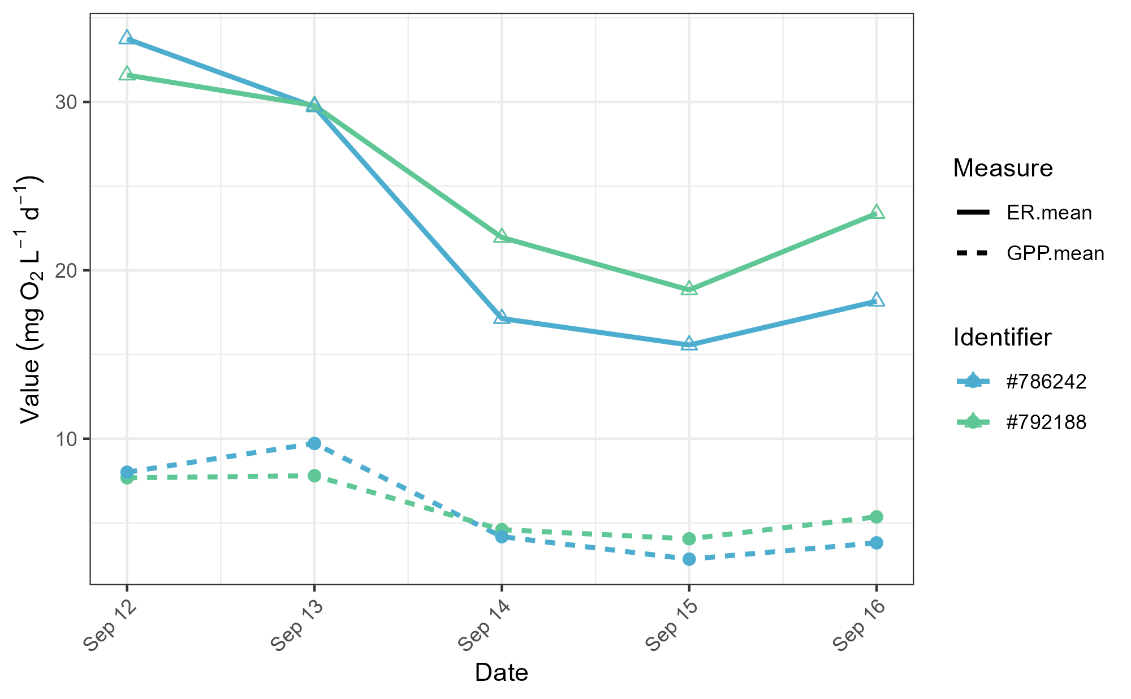


S1.2 Fig. Time series plot for daily average GPP and ER calculated from MiniDOT #786242 and MiniDOT #92188 measurements.
