# Supplementary material for: Environmental drivers of stream metabolism in a middle TN headwater stream
Source: PLoS One. 2024 Dec 31;19(12):e0315978. doi: 10.1371/journal.pone.0315978 (PMC11687656; doi:10.1371/journal.pone.0315978)
Supplement: S2 File — (DOCX) [file pone.0315978.s002.docx]

## S2 Site Result Comparisons

All the field campaigns were conducted at site 1 until October 2022, after which it was abandoned because of concerns about the equipment security and its proximity to Stephens Lake in the upper stream. Metabolism results from Site 1, 1.2 km downstream from the lake body, with low flow velocity may retain some characteristics of the lake rather than the stream [1]. Beginning in October 2022, all the data was collected only at Site 2.

To test the validity of prior data collected at Site 1, one field experiment was made in October 2022 to make simultaneous measurements at Sites 1 and 2. On October 25, the DO measurement at Site 1 was abnormally low, with a daily average of only 3.2 mg/L, and did not show a strong diel signal (Fig S2.1). It is possible that the sensor was partly blocked or oriented differently on that day. Procedures were adjusted to prevent similar issues in later measurements. The low DO measurement on October 25 may also have led to the high ER and K_O2_ values in BASEmetab model output for Site 1 on that day. The other two days showed almost no variation in GPP and ER compared with Site 1 and Site 2 (Fig S2.1). The Shapiro-Wilk normality test showed that instantaneous measurements of GPP, ER, and K_O2_ at each site were not normally distributed, so the nonparametric Wilcoxon signed rank test was used to show that Sites 1 and 2 had statistically significant differences (p < 0.05) in time-paired instantaneous values of GPP, ER, and K. However, instantaneous values of GPP, ER, and K at the two sites are highly correlated (p values always < 0.05 for the null hypothesis that the correlation is zero, Spearman r values are GPP = 0.98, ER = 0.998, K_O2_ = 0.998), so we concluded that our measurements at Site 1 should be representative of stream metabolism. Of the 14,688 sets of instantaneous measurements 4,608 (31%) were made at Site 1, including all measurements before October 2022.


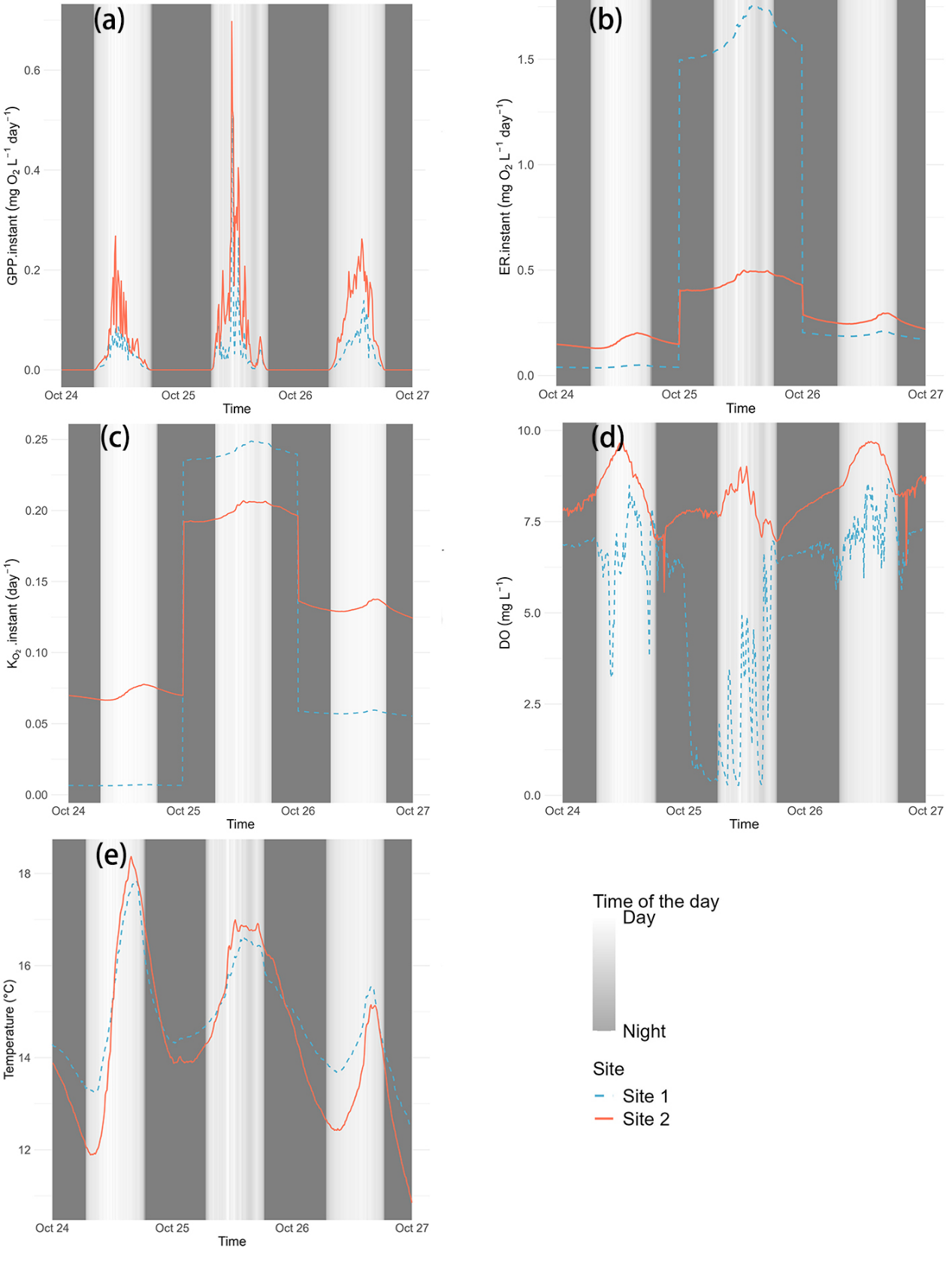


Fig. S2.1 Time series plots for instantaneous values of GPP, ER, K_O2_, DO, and temperature at Sites 1 and 2 from Oct 24 – 26, 2022. The white shading in the background represents daytime and the gray shading represents nighttime.
