# Supplementary material for: Environmental drivers of stream metabolism in a middle TN headwater stream
Source: PLoS One. 2024 Dec 31;19(12):e0315978. doi: 10.1371/journal.pone.0315978 (PMC11687656; doi:10.1371/journal.pone.0315978)
Supplement: S3 File — (DOCX) [file pone.0315978.s003.docx]

## S3 Water Chemistry, July 2022


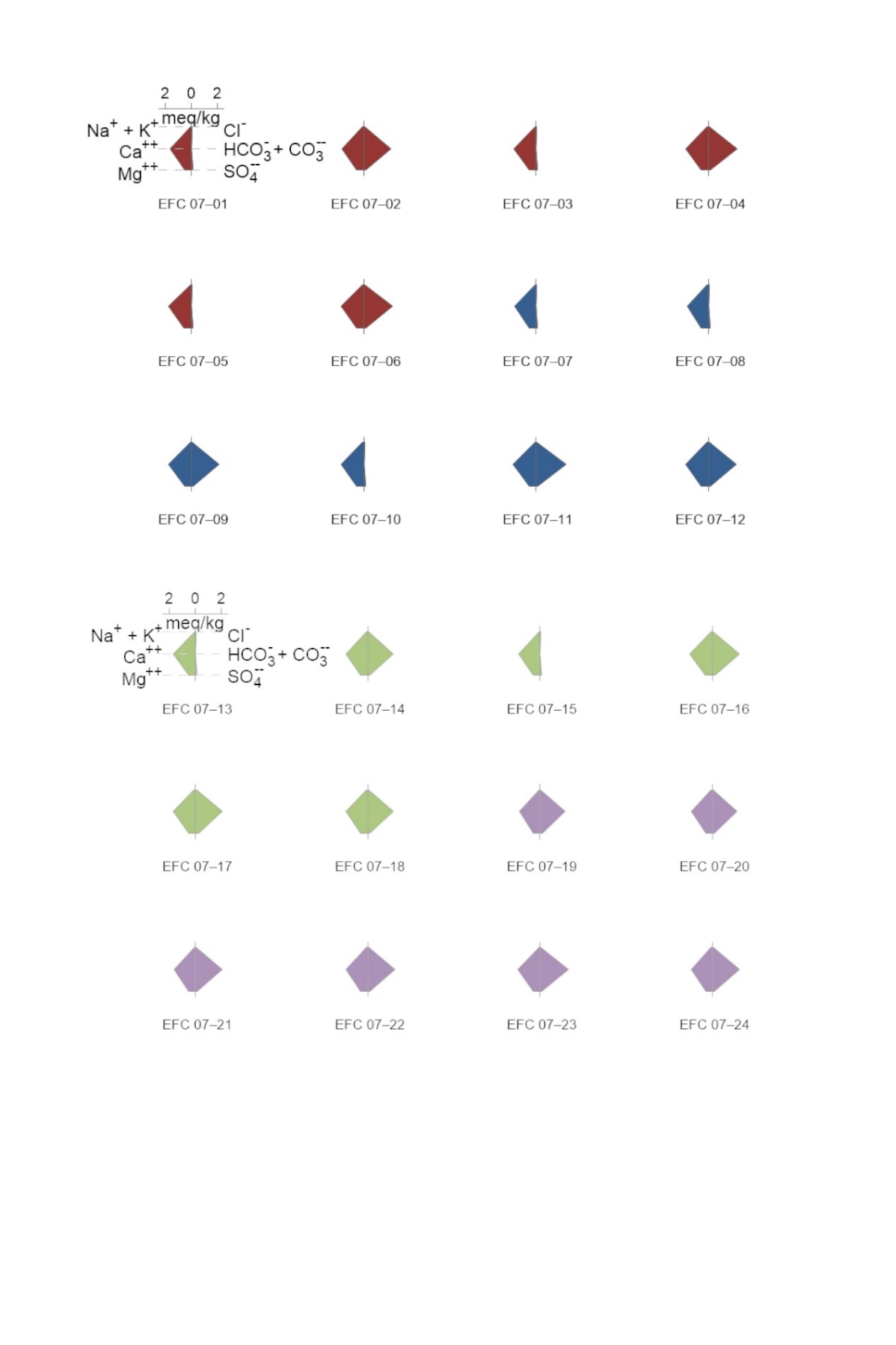


S3.1 Fig. Stiff diagrams including major water species for water samples collected every four hours from July 2^nd^ to 5^th^, 2022. Radial plots with the same color represents the water sample collected from the same day. Samples from EFC 07-01 to EFC 07-06 were colleced on July 2^nd^; samples from EFC 07-07 to EFC 07-12 were collected on July 3^rd^; samples from EFC 07-13 to EFC 07-18 werer colleced on July 4^th^; samples from EFC 07-19 to EFC 07-24 were collected on July 5^th^.

| **Sample** | **DateTime** | **Temp °C** | **Light lux** | **Chl RFU** | **Depth m** | **fDOM RFU** | **ODO % sat** | **ODO % loc** | **SpCond µS cm^-1^** | **TAL PC RFU** | **TDS mg L^-1^** | **pH** | **ODO mg L^-1^** |
| --- | --- | --- | --- | --- | --- | --- | --- | --- | --- | --- | --- | --- | --- |
| EFC 07-01 | 7/2/2022 0:00 | 21.87 | 0 | 0.23 | 0.33 | 7.96 | 67 | 68 | 250.6 | 0.04 | 163 | 7.72 | 5.83 |
| EFC 07-02 | 7/2/2022 4:00 | 21.12 | 0 | 0.24 | 0.327 | 8.13 | 67 | 68 | 250.2 | 0.11 | 163 | 7.73 | 5.95 |
| EFC 07-03 | 7/2/2022 8:00 | 21.10 | 3404 | 0.23 | 0.345 | 7.81 | 77 | 79 | 253.4 | 0.09 | 165 | 7.79 | 6.86 |
| EFC 07-04 | 7/2/2022 12:00 | 24.81 | 44380 | 0.21 | 0.34 | 6.73 | 121 | 123 | 249.1 | -0.12 | 162 | 8.13 | 10 |
| EFC 07-05 | 7/2/2022 16:00 | 26.61 | 18842 | 0.19 | 0.321 | 6.84 | 138 | 141 | 243 | -0.27 | 158 | 8.43 | 11.1 |
| EFC 07-06 | 7/2/2022 20:00 | 24.30 | 227 | 0.21 | 0.325 | 7.71 | 92 | 94 | 246.5 | -0.12 | 160 | 7.99 | 7.73 |
| EFC 07-07 | 7/3/2022 0:00 | 22.37 | 0 | 0.29 | 0.345 | 8.57 | 75 | 76 | 235.3 | 0.04 | 153 | 7.81 | 6.49 |
| EFC 07-08 | 7/3/2022 4:00 | 21.91 | 0 | 0.26 | 0.346 | 9.25 | 66 | 67 | 250.4 | 0.01 | 163 | 7.64 | 5.8 |
| EFC 07-09 | 7/3/2022 8:00 | 22.07 | 5245 | 0.27 | 0.368 | 10 | 78 | 79 | 247.2 | 0.04 | 161 | 7.7 | 6.82 |
| EFC 07-10 | 7/3/2022 12:00 | 25.12 | 44257 | 0.23 | 0.368 | 8.16 | 121 | 123 | 238.9 | -0.11 | 155 | 8.1 | 10 |
| EFC 07-11 | 7/3/2022 16:00 | 27.02 | 3910 | 0.19 | 0.351 | 8.03 | 119 | 121 | 233 | -0.25 | 151 | 8.14 | 9.46 |
| EFC 07-12 | 7/3/2022 20:00 | 24.05 | 112 | 0.23 | 0.355 | 8.76 | 88 | 89 | 232.3 | -0.04 | 151 | 7.88 | 7.37 |
| EFC 07-13 | 7/4/2022 0:00 | 22.60 | 0 | 0.28 | 0.371 | 8.82 | 67 | 68 | 240.1 | 0.05 | 156 | 7.69 | 5.78 |
| EFC 07-14 | 7/4/2022 4:00 | 21.88 | 0 | 0.24 | 0.357 | 8.68 | 67 | 68 | 245.6 | 0.07 | 160 | 7.7 | 5.88 |
| EFC 07-15 | 7/4/2022 8:00 | 21.62 | 2821 | 0.26 | 0.375 | 8.23 | 79 | 80 | 247.8 | 0.08 | 161 | 7.77 | 6.95 |
| EFC 07-16 | 7/4/2022 12:00 | 24.72 | 32195 | 0.23 | 0.375 | 7.38 | 121 | 123 | 243.4 | -0.17 | 158 | 8.15 | 10.08 |
| EFC 07-17 | 7/4/2022 16:00 | 28.81 | 12349 | 0.22 | 0.345 | 6.74 | 140 | 143 | 240.4 | -0.4 | 156 | 8.32 | 10.81 |
| EFC 07-18 | 7/4/2022 20:00 | 24.84 | 156 | 0.21 | 0.35 | 8 | 86 | 88 | 242.4 | -0.11 | 158 | 7.88 | 7.16 |
| EFC 07-19 | 7/5/2022 0:00 | 22.75 | 0 | 0.25 | 0.35 | 8.29 | 66 | 67 | 244.5 | 0.02 | 159 | 7.68 | 5.65 |
| EFC 07-20 | 7/5/2022 4:00 | 22.20 | 0 | 0.26 | 0.35 | 8.44 | 66 | 67 | 248.2 | 0.05 | 161 | 7.71 | 5.71 |
| EFC 07-21 | 7/5/2022 8:00 | 21.98 | 2975 | 0.26 | 0.364 | 8.07 | 76 | 77 | 252.4 | 0.05 | 164 | 7.77 | 6.63 |
| EFC 07-22 | 7/5/2022 12:00 | 25.53 | 9782 | 0.23 | 0.351 | 6.93 | 120 | 122 | 248.5 | -0.14 | 161 | 8.09 | 9.79 |
| EFC 07-23 | 7/5/2022 16:00 | 29.52 | 11873 | 0.24 | 0.328 | 6.92 | 136 | 138 | 242.1 | -0.37 | 157 | 8.35 | 10.36 |
| EFC 07-24 | 7/5/2022 20:00 | 26.06 | 210 | 0.25 | 0.326 | 8.33 | 94 | 95 | 236.3 | -0.16 | 154 | 7.98 | 7.57 |

| **Sample** | **Ca**  **mg L^-1^** | **Mg mg L^-1^** | **Na mg L^-1^** | **K**  **mg L^-1^** | **Cl**  **mg L^-1^** | **Si**  **mg L^-1^** | **S**  **mg L^-1^** | **P**  **μg L^-1^** | **Sb**  **μg L^-1^** | **As**  **μg L^-1^** | **Ba**  **μg L^-1^** | **Be**  **μg L^-1^** | **Cd**  **μg L^-1^** | **Cr**  **μg L^-1^** | **Cu**  **μg L^-1^** | **Pb**  **μg L^-1^** | **Tl**  **μg L^-1^** | **NO3 μg L^-1^** |
| --- | --- | --- | --- | --- | --- | --- | --- | --- | --- | --- | --- | --- | --- | --- | --- | --- | --- | --- |
| 07-01 | 33 | 6.33 | 1.65 | 0.794 | 2.20 | 3.77 | 2.40 | 22.3 | 0.56 | 1.08 | 24.7 | 0.079 | 0.20 | 0.25 | 22.61 | 0.58 | 0.24 | 27 |
| 07-02 | 33.5 | 6.48 | 1.68 | 0.816 | 2.20 | 3.81 | 2.39 | 26.0 | 0.51 | 0.79 | 25.8 | 0.027 | 0.08 | 0.15 | 6.50 | 0.09 | 0.15 | 23 |
| 07-03 | 33.9 | 6.38 | 1.71 | 0.839 | 2.23 | 3.8 | 2.34 | 19.9 | 0.35 | 0.67 | 26.0 | 0.018 | 0.06 | 0.08 | 5.05 | 0.04 | 0.12 | 47 |
| 07-04 | 34.7 | 6.5 | 1.66 | 0.799 | 2.18 | 3.81 | 2.28 | 15.2 | 0.30 | 0.59 | 25.7 | 0.015 | 0.05 | 0.08 | 4.33 | 0.04 | 0.08 | 56 |
| 07-05 | 35.5 | 6.67 | 1.66 | 0.787 | 2.17 | 3.8 | 2.30 | 17.0 | 0.24 | 0.59 | 25.0 | 0.013 | 0.04 | 0.06 | 3.01 | 0.03 | 0.06 | 46 |
| 07-06 | 34.2 | 6.56 | 1.64 | 0.74 | 2.11 | 3.77 | 2.32 | 14.6 | 0.22 | 0.57 | 24.4 | 0.007 | 0.05 | 0.05 | 2.90 | 0.02 | 0.05 | 27 |
| 07-07 | 32.9 | 6.43 | 1.66 | 0.799 | 2.15 | 3.78 | 2.31 | 20.2 | 0.18 | 0.62 | 24.2 | 0.007 | 0.05 | 0.03 | 2.36 | 0.02 | 0.04 | 11 |
| 07-08 | 32.4 | 6.39 | 1.74 | 0.899 | 2.27 | 3.81 | 2.28 | 6.5 | 0.25 | 0.61 | 24.2 | 0.006 | 0.09 | 0.03 | 3.10 | 0.04 | 0.04 | 46 |
| 07-09 | 34.8 | 6.6 | 1.63 | 0.813 | 2.13 | 3.8 | 2.44 | 16.9 | 0.24 | 0.53 | 26.3 | 0.006 | 0.06 | 0.06 | 3.55 | 0.02 | 0.04 | 65 |
| 07-10 | 35.1 | 6.49 | 1.61 | 0.757 | 2.06 | 3.76 | 2.31 | 7.6 | 0.21 | 0.52 | 24.7 | 0.007 | 0.06 | 0.04 | 3.05 | 0.01 | 0.03 | 67 |
| 07-11 | 35.7 | 6.67 | 1.71 | 0.802 | 2.19 | 3.78 | 2.40 | 16.6 | 0.31 | 0.54 | 25.7 | 0.023 | 0.05 | 0.07 | 3.73 | 0.03 | 0.04 | 66 |
| 07-12 | 34.7 | 6.62 | 1.76 | 0.948 | 2.27 | 3.73 | 2.42 | 21.2 | 0.23 | 0.57 | 24.2 | 0.002 | 0.07 | 0.05 | 3.83 | 0.06 | 0.03 | 53 |
| 07-13 | 33.5 | 6.48 | 1.68 | 0.833 | 2.16 | 3.76 | 2.41 | 20.5 | 0.16 | 0.61 | 23.9 | 0.003 | 0.04 | 0.02 | 2.94 | 0.03 | 0.03 | 42 |
| 07-14 | 34 | 6.65 | 1.74 | 0.74 | 2.19 | 3.8 | 2.47 | 28.2 | 0.16 | 0.52 | 25.6 | 0.002 | 0.05 | 0.05 | 3.83 | 0.05 | 0.02 | 47 |
| 07-15 | 32.8 | 6.2 | 1.61 | 0.969 | 2.16 | 3.57 | 2.29 | 22.1 | 0.16 | 0.49 | 24.6 | 0.003 | 0.04 | 0.02 | 3.62 | 0.01 | 0.02 | 67 |
| 07-16 | 34.9 | 6.6 | 1.71 | 0.897 | 2.25 | 3.75 | 3.08 | 20.6 | 0.20 | 0.76 | 26.5 | 0.007 | 0.05 | 0.06 | 5.05 | 0.11 | 0.17 | 117 |
| 07-17 | 34.1 | 6.5 | 2.52 | 1.45 | 3.20 | 3.47 | 3.63 | 40.7 | 0.20 | 0.67 | 25.5 | 0.001 | 0.06 | 0.06 | 11.24 | 0.16 | 0.09 | 130 |
| 07-18 | 32.9 | 6.29 | 1.63 | 0.826 | 2.18 | 3.57 | 2.76 | 16.1 | 0.17 | 0.58 | 23.3 | 0.003 | 0.05 | 0.03 | 3.10 | 0.04 | 0.06 | 68 |
| 07-19 | 31.8 | 6.12 | 1.60 | 0.808 | 2.13 | 3.62 | 2.56 | 13.1 | 0.13 | 0.57 | 23.1 | 0.003 | 0.03 | 0.04 | 2.63 | 0.03 | 0.05 | 48 |
| 07-20 | 31.9 | 6.13 | 1.62 | 0.803 | 2.14 | 3.67 | 2.44 | 18.9 | 0.11 | 0.62 | 24.1 | 0.002 | 0.06 | 0.04 | 2.40 | 0.02 | 0.04 | 49 |
| 07-21 | 33 | 6.24 | 1.63 | 0.826 | 2.16 | 3.69 | 2.36 | 14.2 | 0.20 | 0.58 | 25.7 | 0.009 | 0.05 | 0.06 | 3.42 | 0.03 | 0.05 | 63 |
| 07-22 | 33.5 | 6.29 | 1.61 | 0.905 | 2.09 | 3.6 | 2.25 | 17.4 | 0.16 | 0.50 | 24.5 | 0.001 | 0.04 | 0.03 | 3.17 | 0.03 | 0.03 | 60 |
| 07-23 | 34 | 6.43 | 1.53 | 0.756 | 1.98 | 3.63 | 2.17 | 11.5 | 0.15 | 0.48 | 24.6 | 0.004 | 0.04 | 0.03 | 2.91 | 0.04 | 0.03 | 48 |
| 07-24 | 32.7 | 6.24 | 1.58 | 0.8 | 2.10 | 3.56 | 2.27 | 11.9 | 0.23 | 0.51 | 23.2 | 0.001 | 0.06 | 0.04 | 3.66 | 0.02 | 0.03 | 39 |

S3.1 Table. Summary of all measured species concentrations for water samples collected at EFC from July 2nd to July 5th, 2022
