# Supplementary material for: Environmental drivers of stream metabolism in a middle TN headwater stream
Source: PLoS One. 2024 Dec 31;19(12):e0315978. doi: 10.1371/journal.pone.0315978 (PMC11687656; doi:10.1371/journal.pone.0315978)
Supplement: S4 File — (DOCX) [file pone.0315978.s004.docx]

## S4 Time series plots for July 2022 discrete measurements


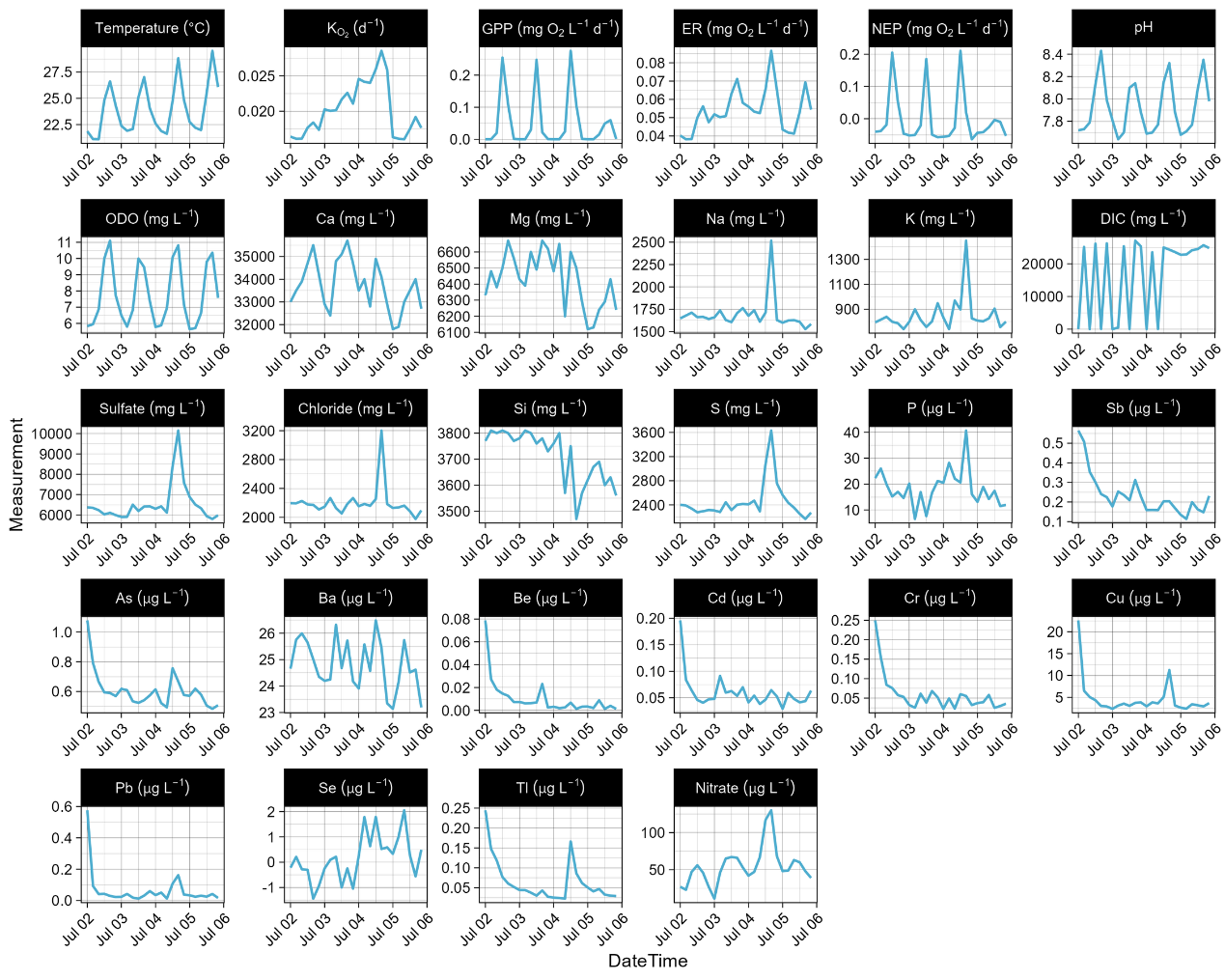


S4.1 Fig. Time series for discrete measurements from EXO_2_ and water samples in EFC. The measurements were made, and samples collected every four hours from July 2^nd^ to 6^th^, 2022.


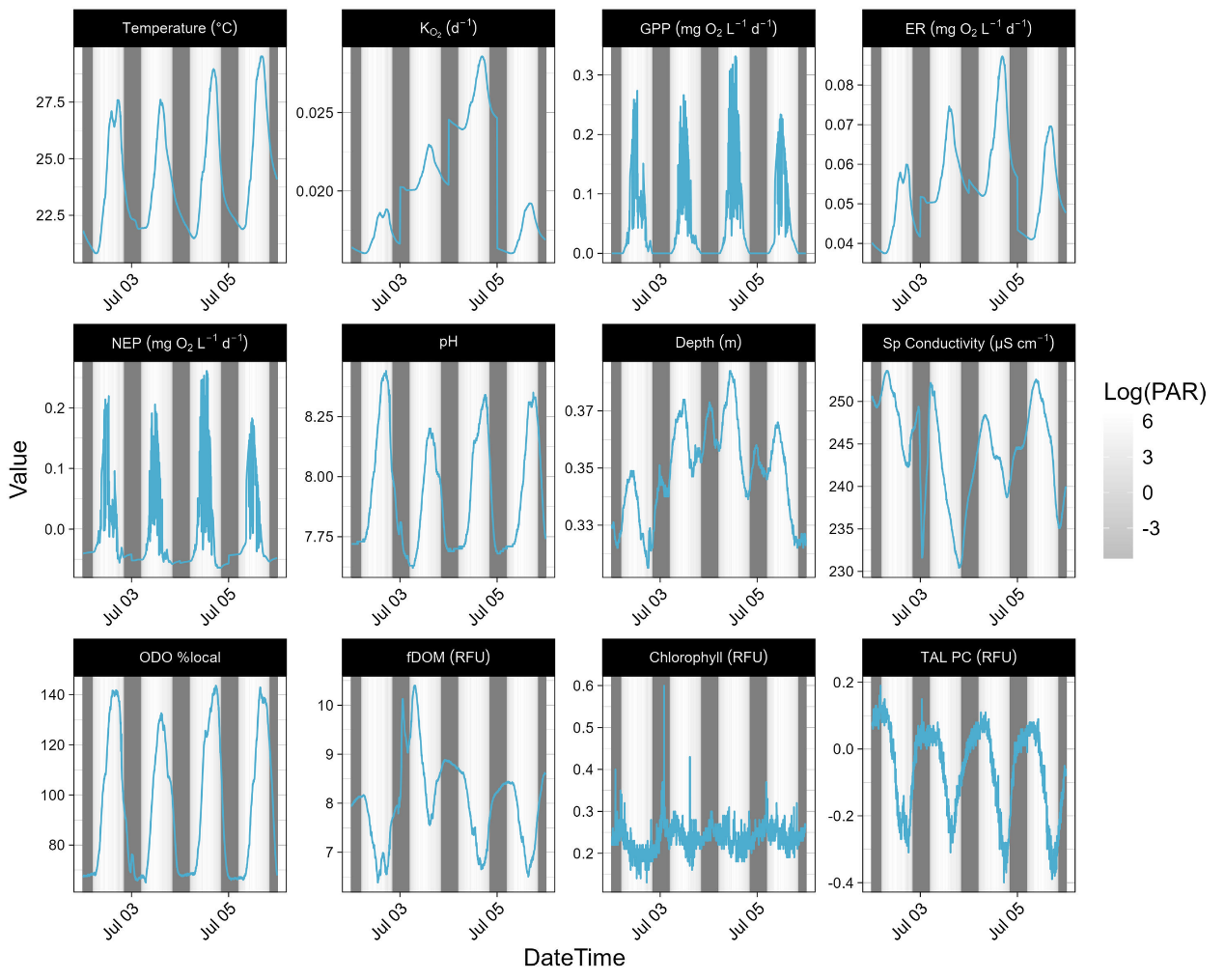


S4.2 Fig. Time Series for continuous measurements from EXO_2_ in EFC. The measurements were made every five minutes from July 2^nd^ to 6^th^, 2022.
