# Supplementary material for: Environmental drivers of stream metabolism in a middle TN headwater stream
Source: PLoS One. 2024 Dec 31;19(12):e0315978. doi: 10.1371/journal.pone.0315978 (PMC11687656; doi:10.1371/journal.pone.0315978)
Supplement: S5 File — (DOCX) [file pone.0315978.s005.docx]

## S5 Correlation matrices

Data collected from July 2nd to July 5th, 2022 at EFC Site 1. Spearman correlation analysis was used to determine the correlation matrix and statistically insignificant values (p > 0.05) were removed for both discrete and continuous measurements (Figures S5.1 & S5.2). GPP was found to be positively correlated with pH, DO, light intensity, concentration of Ca, and nitrate, and negatively correlated with DOM and TAL. ER is found to be positively correlated with temperature, pH, DO, and negatively correlated with conductivity and TAL (Figures S5.1 & S5.2).


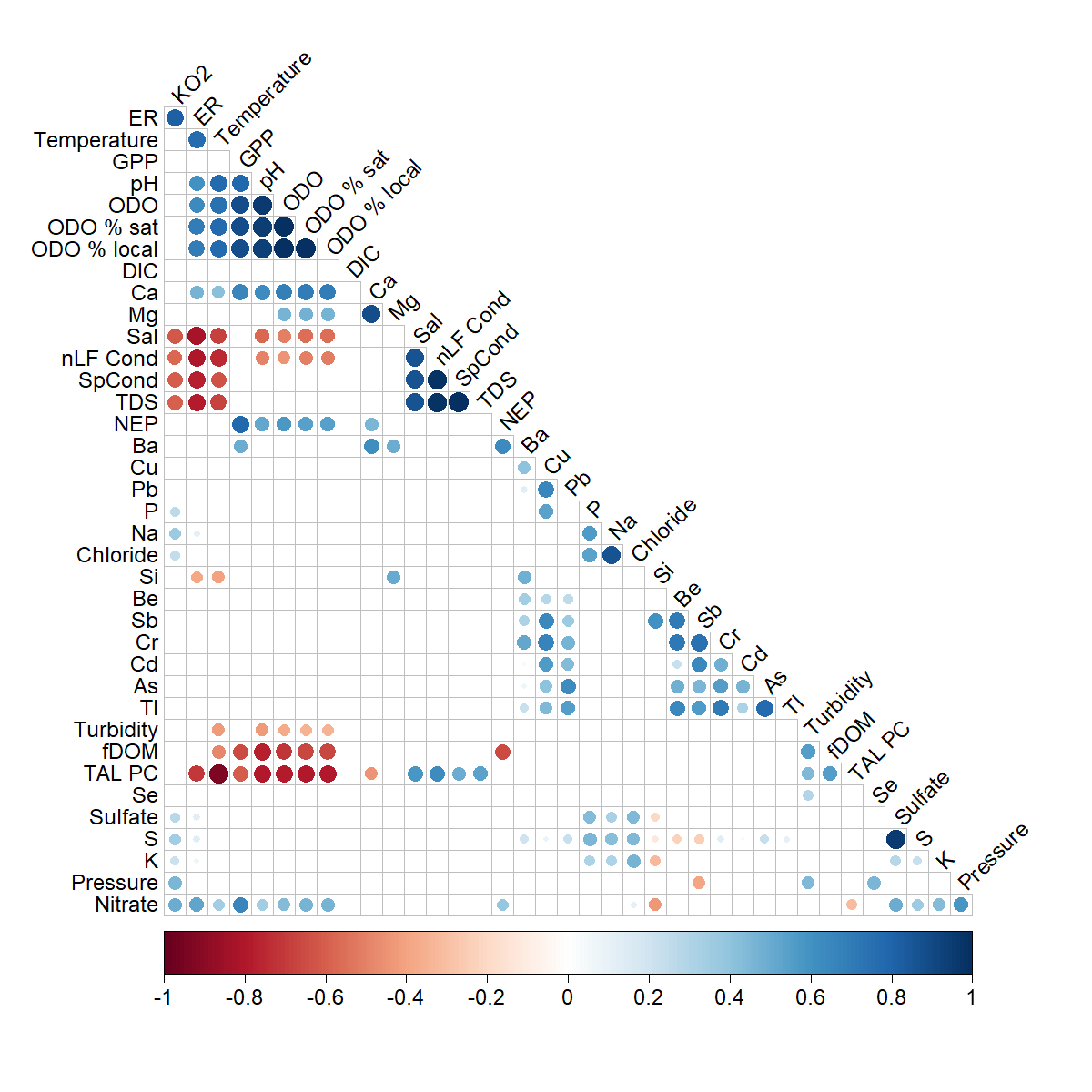


S5.1 Fig. Correlation matrix for discrete measurements in EFC. Boxes for statistically insignificant values (p > 0.05) were left blank. The size and color for the dots represent spearman’s rho values.


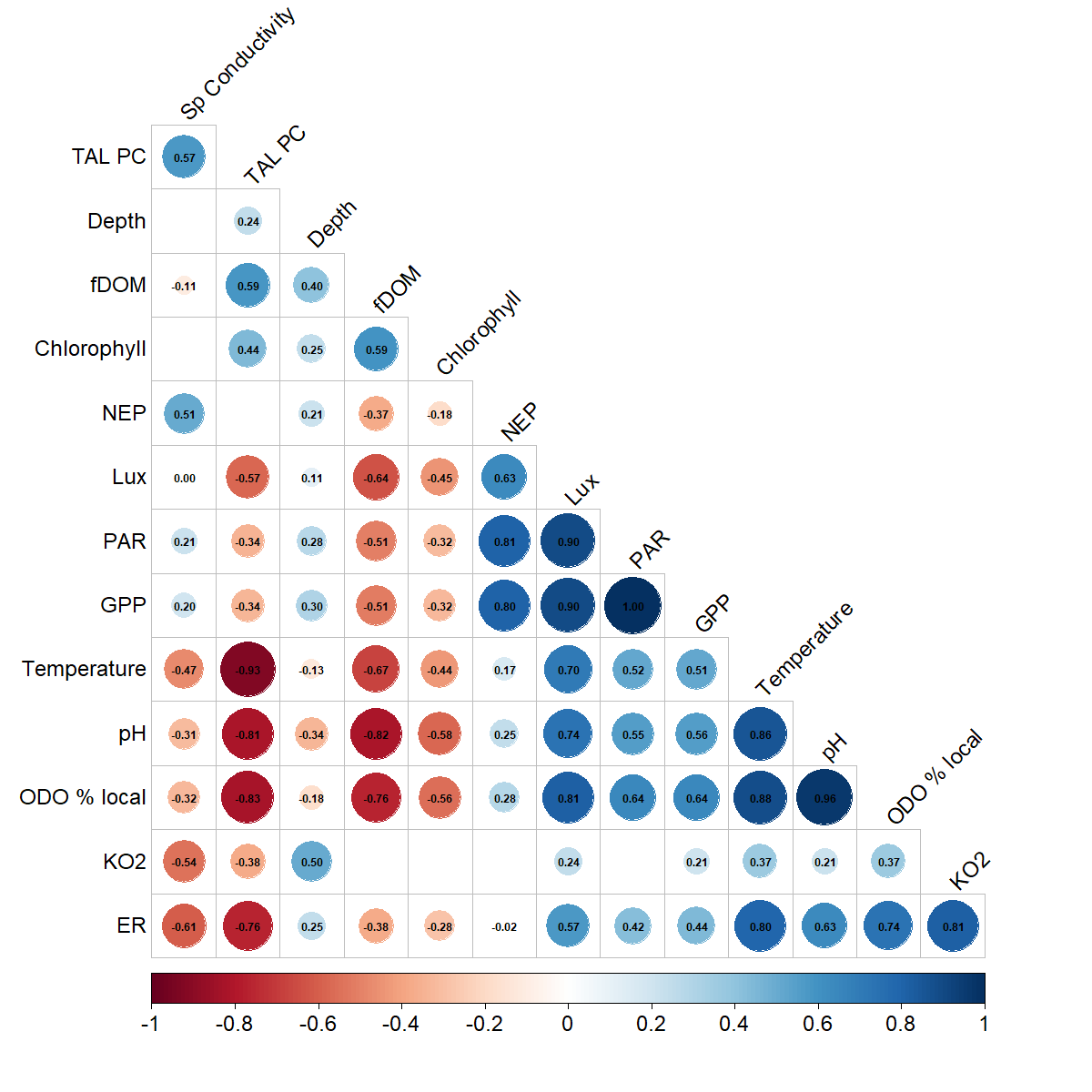


S5.2 Fig. Correlation matrix for continuous measurements in EFC. Boxes for statistically insignificant values (p > 0.05) were left blank. Spearman correlation coefficients were labeled for statistically significant values.
