# Supplementary material for: Environmental drivers of stream metabolism in a middle TN headwater stream
Source: PLoS One. 2024 Dec 31;19(12):e0315978. doi: 10.1371/journal.pone.0315978 (PMC11687656; doi:10.1371/journal.pone.0315978)
Supplement: S6 File — (DOCX) [file pone.0315978.s006.docx]

## S6 Measured DO vs Predicted DO

The scaling model of stream DO concentrations established by Abdul-Aziz & Gebreslase [1] shows that:

$$\begin{aligned} DO = {10}^{18.94}\cdot T_{w}^{-7.46}\cdot pH^{0.45} \# \end{aligned}$$

where DO is dissolved oxygen concentration (mg/l) and T_w_ is water temperature (K). Comparing the model-predicted values and the measured DO concentrations from July 2nd to July 5th, 2022, we found a strong correlation (R^2^ = 0.76) between measured and predicted concentration but not a 1:1 relationship.


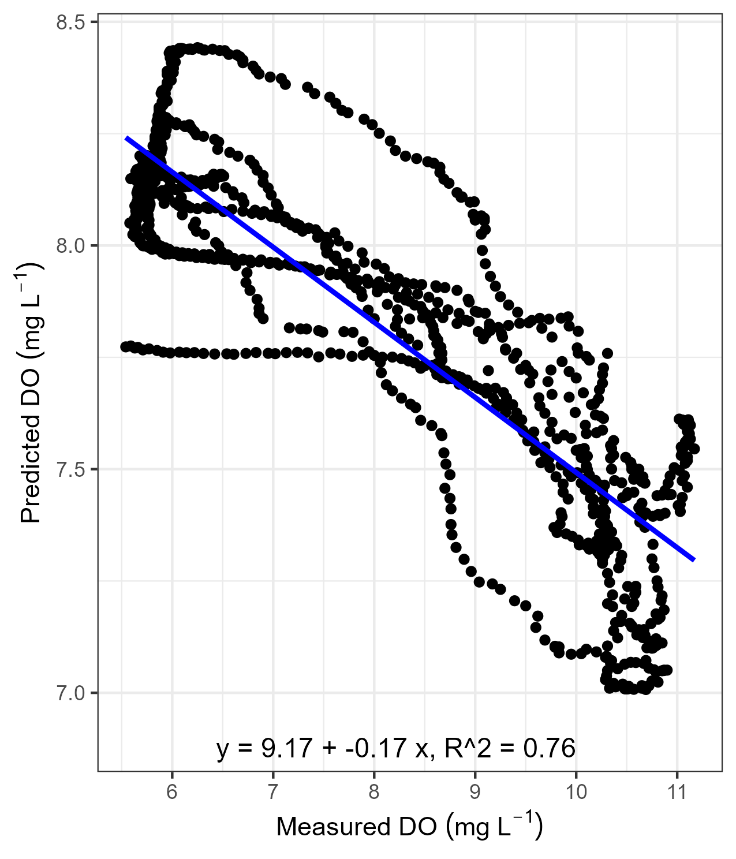


S6.1 Fig. Measured DO vs Predicted DO. Measurements were made from July 2nd to July 5th, 2022, in EFC. The blue line is the best linear fit for the datapoints with the equation at the bottom of the graph.

**References**

1. Abdul-Aziz OI, Gebreslase AK. Emergent Scaling of Dissolved Oxygen (DO) in Freshwater Streams Across Contiguous USA. Water Resour Res. 2023;59. doi:10.1029/2022WR032114
