# Supplementary figures and images for: Environmental drivers of stream metabolism in a middle TN headwater stream
Source: PLoS One. 2024 Dec 31;19(12):e0315978. doi: 10.1371/journal.pone.0315978 (PMC11687656; doi:10.1371/journal.pone.0315978)

## S7 Sample BASEmetab output for July 4, 2022 at East Fork Creek site 1


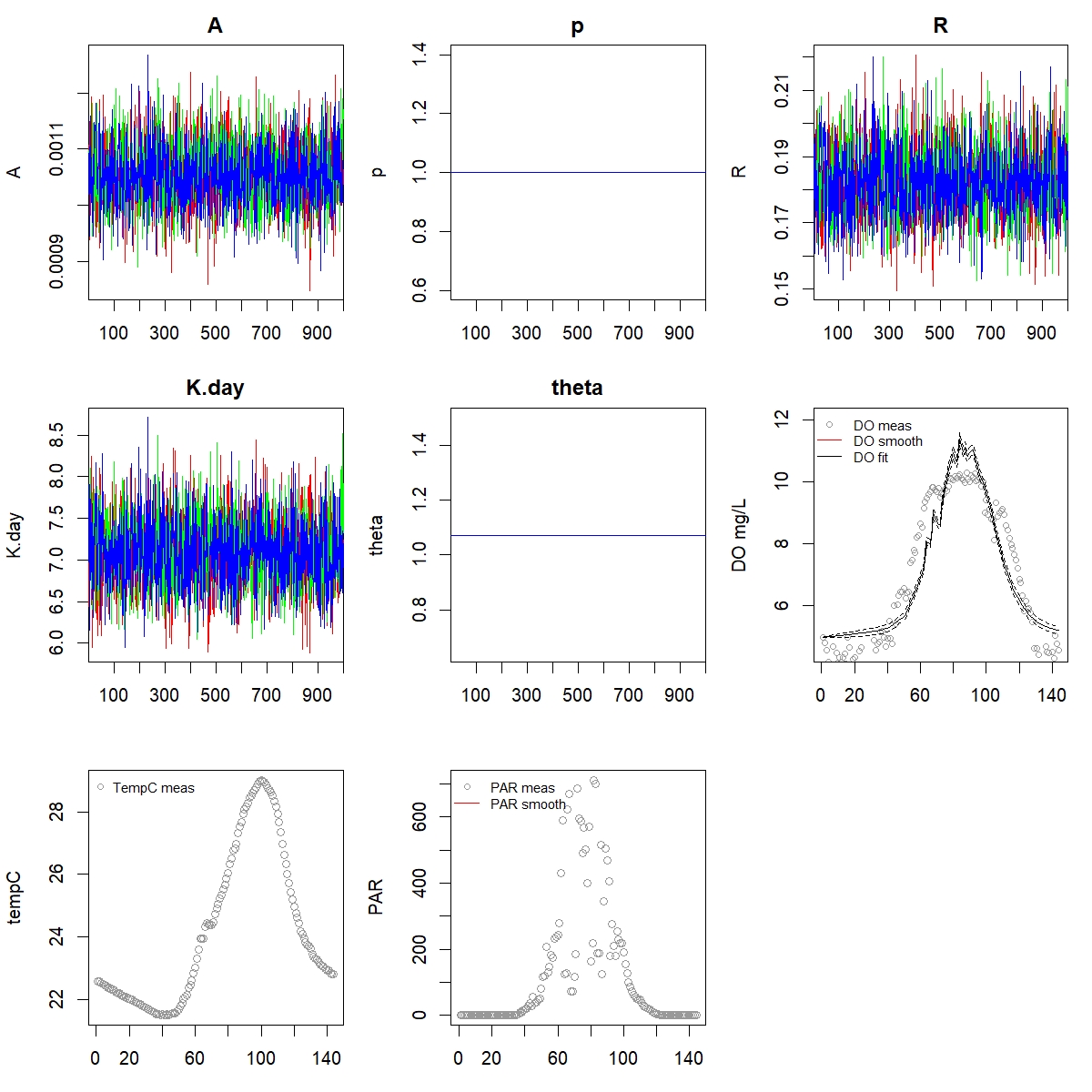


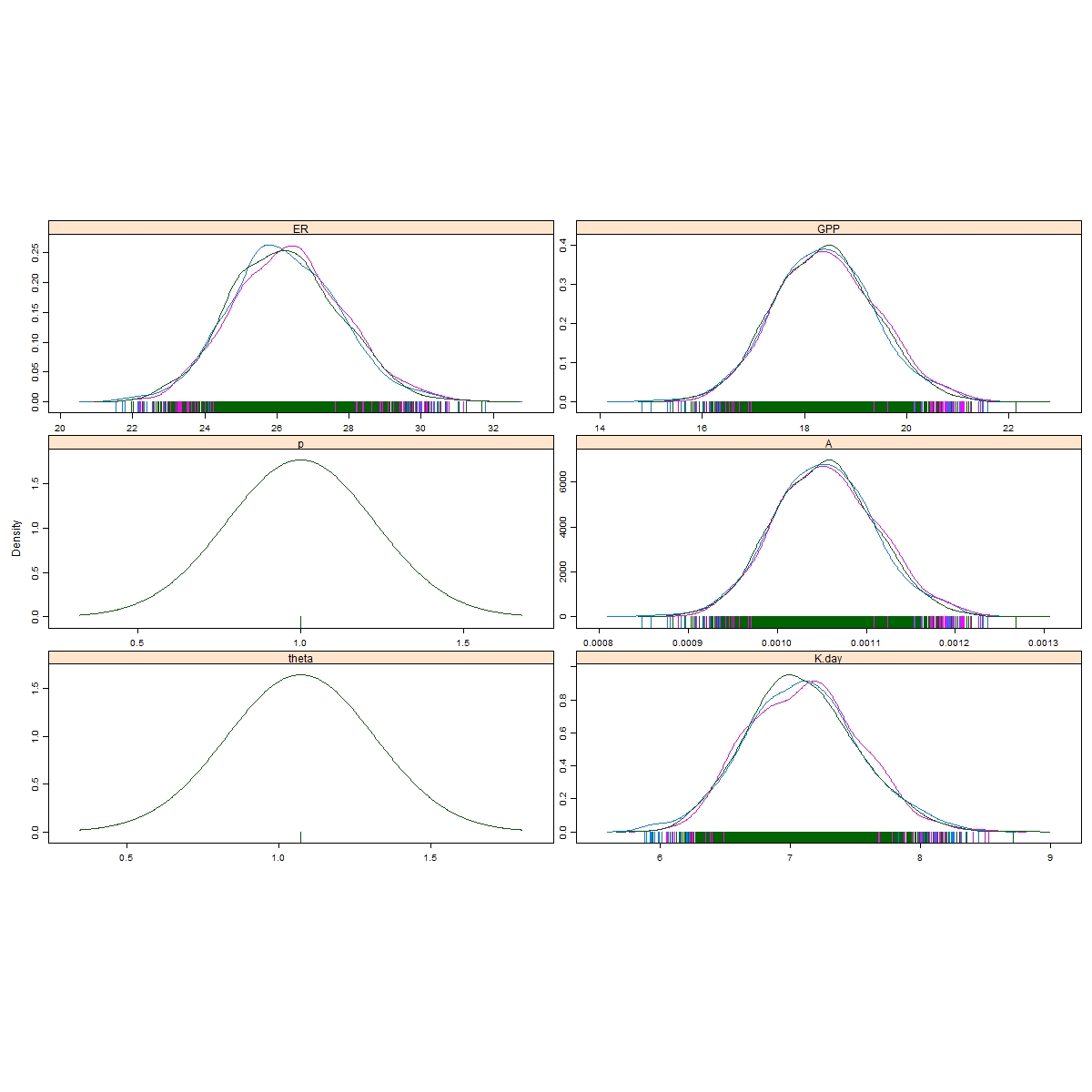

Supplement: S7 File — (DOCX) [file pone.0315978.s007.docx]
