# Supplementary material for: Environmental drivers of stream metabolism in a middle TN headwater stream
Source: PLoS One. 2024 Dec 31;19(12):e0315978. doi: 10.1371/journal.pone.0315978 (PMC11687656; doi:10.1371/journal.pone.0315978)
Supplement: S8 File — (DOCX) [file pone.0315978.s008.docx]

## S8 Instructions for stream metabolism measurements

**Essential equipment and supplies for each site**

1. HOBO Pendant® MX Temperature/Light Data Loggers
2. HOBO U20L pressure loggers, one for air and one for water level
3. miniDOT and miniwiper
4. Paracord

**Optional equipment**

- YSI Exo1 to measure salinity (used to calculate DO solubility)

**Pre-trip checklist**

- Charge batteries in all handhelds
- Delete old data files from LI-COR handheld
- Charge portable batteries
- Synchronize time on all handhelds and sensors
- Test disposable batteries (measure voltage with multimeter or handheld)
- Use lens cleaning cloths to clean measurement windows on miniDOTs and HOBO light loggers.
- Calibrate YSI EXO1 sonde
- Use KorEXO software. If KorEXO says that parameter does not require calibration verify by measuring the standard. If the reading is not close to the standard value then recalibrate.
- Pack waterproof covers for handhelds.
- When measuring stream metabolism, make sure to include a sensor to measure atmospheric pressure in addition to DO and temperature (miniDOT) and light intensity (preferably PAR sensor, otherwise HOBO MX2202)
- Start logging on HOBO loggers (light and pressure). Have a backup for each
- If we are using the ISCO autosampler pack it and all of its parts, plus a tent
- Pack a plastic container with the following:
- Water sample bottles + filters and syringe
- Two ripcords
- Sharpie
- Field notebook

**Field experiment preparation**

- To set up logging on pressure sensors:
  - Unscrew and remove cap from each pressure sensor to expose optical window
  - Connect sensor to USB port on lab notebook computer using the HOBO Optical USB base station.
  - Launch HOBOware Pro on computer, Connect to sensor, verify date and time is correct, and configure sensor to start logging on save or at specified datetime with a logging interval of 5 minutes
- To configure HOBO Pendant loggers
  - Install the HOBOconnect app on your phone
  - Press the button on the pendant logger
  - Launch the HOBOconnect app and select the logger to configure
  - Verify date and time is correct and configure sensor to start logging on save or at specified datetime with a logging interval of 5 minutes.
- To configure miniDOT and miniwiper
  - Unscrew and remove cover
  - Connect to computer using USB-C to USB cable
  - File manager should open and show files
  - Launch the javascript file miniDOTControl.jar
  - Verify date and time is correct.
  - Set the logging interval for 5 minutes
- Mount miniDOT and miniwiper onto bracket attachment
- If using the LI-COR PAR sensor with the LI-1500 Light Sensor Logger
  - Mount sensor to 2009S Lowering Frame using insulating parts kit
  - Connect sensor to logger using cable
  - Turn logger on, verify date and time are correct.
  - Choose the "EFC" configuration file
  - Set the logging interval at 5 minutes
  - Connect a charged portable Lithium battery to the logger
  - Tie/clamp the frame to the dock or mooring
  - Place the logger and battery inside a waterproof container
- To calibrate the YSI Exo1
  - Connect the sonde to the lab notebook computer USB port using the USB signal output adapter + cable
  - Launch the KorEXO software and press "connect" in the Instrument connection panel
  - For each sensor, if KorEXO says that parameter does not require calibration verify by measuring the standard. If the reading is not close to the standard value then recalibrate.

**Field site**

When placing and retrieving sensors:

- Record time
- Record GPS location
- Take photo
- Note whether sensors have become fouled or displaced
- Record salinity
- Collect filtered water sample

**Post-field experiment**

- Place labeled water sample bottles in refrigerator
- Set wet materials such as paracord out to dry in SC5712
- Turn off miniDOT, miniwiper, and HOBO loggers and download data
- Use ExoKOR software to turn off logging and download data. Note: all data files should be saved to our SharePoint site.
- **MiniDOT**: Remove sleeve, turn logging off, connect to PC, open the folder and delete old files, then run "concatenate.jar" to create a single output file. You will have to input the measured salinity in ppt and either the elevation in meters or the atmospheric pressure in atmospheres. Remove the headers from the Cat.txt file.
- **HOBO light logger**: Use HOBOconnect app on your phone to download the data to your phone as an Excel file, then email the file to yourself. In the data file multiply the light intensity (lux) by 0.0185 to obtain PPFD (photosynthetic photon flux density). Or use the average ratio LI-COR PAR/HOBO_Raw for the specific sensor.
- **Pressure logger**: unscrew the top and attach the logger to the HOBO USB station. Open the HOBOware App on laptops. Stop, readout data, plot, then export the csv data file to the data folder. Export pressure in atmospheres.
- **Par sensor**: download the txt data file from the folder and add file to data folder.

**Initial setup for data analysis**

1. Download and install R and Rstudio from [RStudio Desktop - Posit](https://posit.co/download/rstudio-desktop/)
2. Go to [johncayers/StreamMetabolismBasemetab: Script for running BASEmetab package of Grace 2015. (github.com)](https://github.com/johncayers/StreamMetabolismBasemetab)
3. Choose Code: Download ZIP, then extract folder and contained files to Documents folder
4. In RStudio open the project file. This will install renv
5. Type "library(renv)"
6. Type renv::restore() to install packages
7. You also must install the program the program JAGS: see [JAGS - Just Another Gibbs Sampler (sourceforge.io)](https://mcmc-jags.sourceforge.io/). I downloaded and installed version 4.3 from <https://sourceforge.net/projects/mcmc-jags/files/JAGS/4.x/Windows/>

**Processing data**

- Combine data from MiniDOT and HOBO output files by matching the timestamps as closely as possible. You should end up with a csv file with DO is mg/L, pressure in atmospheres, and salinity in parts per thousand ppt. This is the format for input into BASEmetab.
- Open the R file called StreamMetab, manually click the three packages on the right window,
- Make sure to change "setwd" to the current working directory by copying the path to the StreamMetab folder before running the code
- Copy and paste all the files in the output folder after running the code into the corresponding folder in SharePoint.
