# Supplementary material for: Environmental drivers of stream metabolism in a middle TN headwater stream
Source: PLoS One. 2024 Dec 31;19(12):e0315978. doi: 10.1371/journal.pone.0315978 (PMC11687656; doi:10.1371/journal.pone.0315978)
Supplement: Attachment — Submitted filename: Response to Reviewers.docx [file pone.0315978.s009.docx]

We thank the reviewers for their helpful suggestions to improve the manuscript. We have made extensive revisions to the manuscript to address the reviewer’s concerns. All figures were revised, and Figure 1 was replaced with a map based on public domain data from the USGS.

Reviewers' comments:

Reviewer's Responses to Questions

**Comments to the Author**

Our replies to each comment in section 5 should address the concerns expressed in the “No” responses of reviewer #2 to questions 1, 2, and 4.

1. Is the manuscript technically sound, and do the data support the conclusions?

 The manuscript must describe a technically sound piece of scientific research with data that supports the conclusions. Experiments must have been conducted rigorously, with appropriate controls, replication, and sample sizes. The conclusions must be drawn appropriately based on the data presented.

Reviewer #1: Partly

Reviewer #2: No

2. Has the statistical analysis been performed appropriately and rigorously?

Reviewer #1: Yes

Reviewer #2: No

3. Have the authors made all data underlying the findings in their manuscript fully available?

The [PLOS Data policy](https://nam04.safelinks.protection.outlook.com/?url=http%3A%2F%2Fc05y1x9s.r.us-east-2.awstrack.me%2FL0%2Fhttp%3A%252F%252Fwww.plosone.org%252Fstatic%252Fpolicies.action%2523sharing%2F1%2F010f0192330dc35d-164b5039-ab7a-4252-a531-336c547df731-000000%2FEndCAxkWh3T8v3L_xxayNls6l6I%3D178&data=05%7C02%7Cjohn.c.ayers%40vanderbilt.edu%7Cfe05a41cfb444868f41908dcdee0420e%7Cba5a7f39e3be4ab3b45067fa80faecad%7C0%7C0%7C638630303813157767%7CUnknown%7CTWFpbGZsb3d8eyJWIjoiMC4wLjAwMDAiLCJQIjoiV2luMzIiLCJBTiI6Ik1haWwiLCJXVCI6Mn0%3D%7C0%7C%7C%7C&sdata=fobVs1JoDcQzPfy1ADzCQrKJbfLVhKHn2TaH0s9hDxU%3D&reserved=0) requires authors to make all data underlying the findings described in their manuscript fully available without restriction, with rare exception (please refer to the Data Availability Statement in the manuscript PDF file). The data should be provided as part of the manuscript or its supporting information, or deposited to a public repository. For example, in addition to summary statistics, the data points behind means, medians and variance measures should be available. If there are restrictions on publicly sharing data—e.g. participant privacy or use of data from a third party—those must be specified.

Reviewer #1: Yes

Reviewer #2: Yes

4. Is the manuscript presented in an intelligible fashion and written in standard English?

PLOS ONE does not copyedit accepted manuscripts, so the language in submitted articles must be clear, correct, and unambiguous. Any typographical or grammatical errors should be corrected at revision, so please note any specific errors here.

Reviewer #1: Yes

Reviewer #2: No

5. Review Comments to the Author

 Please use the space provided to explain your answers to the questions above. You may also include additional comments for the author, including concerns about dual publication, research ethics, or publication ethics. (Please upload your review as an attachment if it exceeds 20,000 characters)

Reviewer #1: General comments:

This manuscript describes an experimental study investigating headwater stream metabolic

regimes in East Fork Creek, a headwater stream in middle Tennessee. The manuscript is generally well written, and the methods are well described, but I do not believe in this state, the results are novel or transformative enough for PLOS One. I provide some other comments that I hope help the authors streamline the manuscript.

Introduction:

-The first three paragraphs of the introduction describe very high-level general knowledge on stream metabolism. This is unnecessary and can be summarized in a couple of sentences with the appropriate citations. Instead, the introduction should synthesize what we know about how stream metabolism is affected by the controls the authors are interested in testing (e.g., temperature; light intensity, etc) and what knowledge gaps this work is contributing to.

We have extensively revised the Introduction to address these concerns.

Materials and Methods:

-The method described to measure flow velocity is inappropriate for this kind of study, I suggest eliminating the comment. Deleted.

-The sensor precision test contributes little to the manuscript. I suggest removing the section and adding a line under field campaign setup along the lines of : “We confirmed the MiniDOTs and 153 HOBO Pendant MX Temperature/Light Data Loggers serve well for the purpose of this study (XXX in Supporting Information)”. Deleted that section.

-If Site 1 was abandoned it should be simply removed from the study and the Site results comparison section eliminated.

We considered deleting site 1 but decided that would greatly weaken our conclusions due to incomplete seasonal coverage. Instead we moved the “Site Comparison” section to Supporting Information and we made clear in the text and in every figure which site is being discussed or plotted.

Results

-For the section describing seasonal variations, there is many data gaps in figure 4 and the data would be better presented in boxplots separated by season. We replaced Fig 4 with a boxplot as suggested.

General

-Address inconsistencies in significant figures in values throughout the manuscript and in figures. Also, in the text, values of ER, GPP, etc are written with too many significant figures. Made those changes throughout.

Reviewer #2: General comments: This paper aims to investigate seasonal and diurnal variations in metabolism parameters on a headwater stream. To do this the authors measure key environmental parameters at 3 locations within the East Fork Creek. They estimate metabolism utilizing dissolved oxygen data from sensors. The manuscript in its current state requires major revisions.

The current writing of the manuscript includes several results and pieces of information that are not contributing to the main story of the paper and thus the ideas behind these extra pieces of information are not fully developed throughout.

We have extensively rewritten the manuscript to make it more focused.

For example, lines 147-149 mention “Another goal of this study was to test the precision and reliability of new light and dissolved oxygen sensors and see if they can make continuous stream metabolism measurements at a lower cost”, this goal is not directly contributing to the original goals stated on the introduction, if a relationship exists it is not clear. Deleted that line.

Furthermore, results from the metabolic ecological model as well as scaling from equation 4 are not fully folded into the discussions which makes the reader question the need for this extra analysis. See response below.

Additionally, the authors describe 3 key sites for their study but then it seems that the major results are only from one site, it is not clear the need for all the sites or which results come from which of the riverine locations. We have deleted all data for the lake, and we have made clear in the text and figures which site measurements were made at.

Overall, the manuscript readability needs to be significantly improved. I recommend thinking deeply about the goals and questions to explore on this manuscript and ensure that all of the results and data presented are clearly addressing those goals and questions.

It would perhaps help to have a combined results and discussion sections with sub-headers that delineate the connection of each section to the goals and questions. We have reorganized the text but opted to keep separate Results and Discussion sections.

Finally, I have major concerns about how metabolism in the lake was calculated as well as the discrepancy in orders of magnitudes for metabolism metrics across figures. With the current state of the writing, I was not able to discern if there is a justifiable reason for the difference in these magnitudes or if a lake specific methodology was applied for lake metabolism calculations.

Agreed, we should not have included the lake data. It has been deleted.

Specific comments:

42-47 I recommend reviewing Chapter 34 - Stream Metabolism (<https://doi.org/10.1016/B978-0-12-813047-6.00012-7>) and adjusting equations accordingly. ER values now negative throughout, and Equation adjusted.

54-63 Discuss GPP controls in detail, add similar content for ER and NEP. Now discussed in second last paragraph of Introduction.

Please revise the statements in 69-79 and back them up with literature. This paragraph is very low on citations. I am familiar with metabolism literature, and I believe some of the statements made are not supported by published studies. It seems the authors are conflicting metabolism studies (i.e., studies that estimate ER and GPP via measured diel variations of dissolved oxygen in water) and freshwater studies that measure CO2 concentrations. We have rewritten this paragraph and added citations.

95 Seems that sites downstream of the lake might be 2nd order streams. We are unsure, so we delete all references to stream order.

162 Were samples filtered upon returning to the university? If so, how many days did the samples spend not filtered? Made clear that samples were processed immediately after the last sample was collected. Now states the “samples were processed immediately after the last sample collected.”

188-192 Results, rephrase please. Deleted.

223-239 It is unclear why the authors need to estimate metabolism at 2 sites. Seems the questions posed on the instruction could be addresses just by looking at the one station metabolism outputs from Site 2 since it had more robust data and data collected for a longer period of time.

 We considered deleting site 1 but decided that would greatly weaken our conclusions due to incomplete seasonal coverage. Instead we moved the “Site Comparison” section to Supporting Information and we made clear in the text and in every figure which site is being discussed or plotted.

230 How was metabolism at Stephens Lake estimated? A lake doesn’t meet the assumptions for BASEmetab. Agreed, metabolism estimates for Stephens Lake were removed. Lake metabolism results have been removed.

235-236 “instantaneous values of GPP, ER, and K at the two sites are highly correlated (Pearson and Spearman p values always < 0.05 for the null hypothesis that the correlation is zero)” This statement needs reporting of correlation coefficients in addition to p-value. The correlation might be significant but not necessarily strong. The site comparison results have been moved to Supporting Information and correlation coefficients are now reported.

244-247 Unclear on the connection of these sentences with the 242-244 statements. Moved lines 242-244 to the "seasonal variations" section for clarification

250 Is this at site 2? Also, were the DO, HOBO and PAR sensors deployed for 57 days or just 3-7 days as stated in line 128? We originally were reporting 57 total days with 3-7 days per campaign. We have rewritten for clarity.

Please include plots of the time series of the DO data in the SI. We have packaged the input and output files from BASEmetab and uploaded them to Figshare; a doi with url will be included on manuscript resubmission.

Was discharge measured? Since the sites are downstream of the lake, what is the influence that the lake has on the stream discharge and therefore the apparent seasonal patterns? No, discharge was not measured.

Fig 4. Methods need to include descriptions on average calculations or state if this is a model output. This figure is replaced by seasonal boxplots. All reported values of GPP, ER, and K_O2_ are model outputs from BASEmetab.

ER values across multiple figures are positive. ER represents the consumption of DO for which is usually negative, is this the absolute value of ER? If so, please add this information to the methods and the Figure caption. Yes, we were reporting the absolute value; we have clarified throughout the manuscript. ER values are reported as negative values unless we specifically state otherwise.

285-291 Unclear what was done here. What do the authors mean by “caused by the BASEmetab design” and what do the mean with “calculation does not include any information from the previous day”. A feature of the Bayesian modeling is that you rely on information from the previous days to estimate subsequent days. Deleted.

293 Is this site 1 or site 2? This section on "diurnal variations" contains data only from site 1, which we now make clear.

Fig 5: Figures should be placed within the section they are referenced. Was the DO time series data QAQC? If so what type of rules were followed? The DO time series data are not QAQC. They are the primary input to BASEmetab for calculating the stream metabolism parameters.

Metabolism estimates plotted on Fig 5 seem orders of magnitude lower than the averages on Fig 3 and 4, how are they different? That is because they are 5-minute values from BASEmetab. Summing the 5-minute values for a day gives the total daily values for ER, GPP, and K_O2_ - now made clear in the figure caption.

Fig 6, what are the units for Ca, Mg and DIC measurements? Ca and Mg concentrations are in ppb, now stated in figure caption. DIC measurements deleted.

307-309 Needs correlation coefficients and p-values in text. For clarity we report correlation coefficients only for pairs with p < 0.05 and r > 0.5; r values are now reported.

460 Reference style doesn’t match. Fixed.

The discussion doesn’t address all the results presented in the results section. For example, results from the metabolic theory of ecology or the scaling results from equation 4 are not addressed. The metabolic theory of ecology is addressed in the first paragraph of the Ecosystem Respiration section where activation energies are discussed, and in the second paragraph: "Peak ER occurred in the fall at lower mean temperature than the summer. This deviation from predictions of the metabolic theory of ecology can be explained by changes in availability of labile organic carbon." The scaling results from Eq. (4) are briefly addressed: "In EFC, the dominant effect of metabolic activities on diurnal DO variation also causes the deviation from the temperature- and pH-dependent scaling law proposed by Abdul-Aziz & Gebreslase (19)."

It is also not clear how the discussion is answering the questions stated on the introduction as the goal of the paper and how those answers help to “better understand the carbon budget for headwater streams in a future with climate change”. The discussion has been rewritten and reorganized.
