# Supplementary material for: Environmental drivers of stream metabolism in a middle TN headwater stream
Source: PLoS One. 2024 Dec 31;19(12):e0315978. doi: 10.1371/journal.pone.0315978 (PMC11687656; doi:10.1371/journal.pone.0315978)
Supplement: Attachment — Submitted filename: ResponseToReviewersV2.docx [file pone.0315978.s010.docx]

The manuscript was sent back to the two previous reviewers who both unfortunately declined to review the manuscript a second time. Rather than start the review process over with two new reviewers, I have carefully read through your response to the reviewers and the revised manuscript draft. You have done a great job revising the manuscript text in response to each major piece of feedback from the reviewers. I have no further suggestions for the text itself but feel that the figure quality can be improved prior to accepting the manuscript. Here are a few edits I would like you to make in your next revision, mostly related to labels:

Figure 2: The labels appear to be the “raw” text likely used as column labels in your data files. Please clean these up by using sub/superscripts and parentheses for units. For example change GPP_mgO2L-1d-1 to GPP (mg O_2_ L^-1^d^-1^). Likewise, capitalize Measurement. You might consider making the fonts larger as well since I needed to zoom in substantially to read all of the labels. Done.

Figure 3: Same comment as above. Modify all of the labels with super/subscripts and parentheses. In this case listing every date on the x axis is a bit busy, consider only labeling every other day. Done.

Figure 4: Use super/subscripts for the labels Done.

Figure 5: Same comment as above. Done.

Figure 6: Same comment as above for y axis label. Done.

Figure 7: Same comment as above for both axis labels. Done.

Figure 8: Same comment as above for y axis label. Done.

Figure 9: For consistency with other figures change mg/L to mg L^-1^. Consider changing “Values” to “O_2_ concentration” Done.

Figure 10: Same comment as for figure 9 Done.

Figure 11: Same, please use super/sub scripts Done.

Please pay similar attention to the figures and tables in the supporting information file to ensure that they are as high quality as the main text figures and use consistent style (e.g., superscripts for units instead of mg/L) Done.

In addition, we

1. Updated the caption for Figure 5 by adding DO and K_O2_.
2. Fixed the label for S2.1 Figure in the supplement, which was incorrectly labeled as S1.2.
3. In S3.1 Table changed the column names to scientific notation as mentioned by the reviewer to maintain consistency.
